# A Narrative Review of Binge Eating and Addictive Behaviors: Shared Associations with Seasonality and Personality Factors

**DOI:** 10.3389/fpsyt.2013.00183

**Published:** 2013-12-27

**Authors:** Caroline Davis

**Affiliations:** ^1^Kinesiology and Health Sciences, York University, Toronto, ON, Canada

**Keywords:** binge eating, addictive behaviors, seasonality, punishment sensitivity

## Abstract

Binge-eating disorder and seasonal affective disorder were first described as clinically relevant conditions in very close temporal proximity a few decades ago. Both disorders have a higher prevalence rate in woman than in men, are characterized by a high proneness-to-stress and manifest heightened responsiveness to high-calorie, hyper-palatable foods. In recent years, a compelling body of evidence suggests that foods high in sugar and fat have the potential to alter brain reward circuitry in a manner similar to that seen when addictive drugs like alcohol and heroin are consumed in excess. These findings have led to suggestions that some cases of compulsive overeating may be understood as an addiction to sweet, fatty, and salty foods. In this paper, it is proposed that high seasonality is a risk factor for binge eating, especially in those characterized by anxious and impulsive personality traits – associations that could only occur in an environment with a superfluity of, and easy access to, rich and tasty foods. Given the well-established links between binge eating and addiction disorders [Ref. (1–3) for reviews], it is also suggested that seasonality, together with the same high-risk psychological profile, exacerbates the likelihood of engaging in a broad range of addictive behaviors. Data from a community sample (*n* = 412) of adults tested these models using linear regression procedures. Results confirmed that symptoms of binge eating and other addictive behaviors were significantly inter-correlated, and that seasonality, gender, and addictive personality traits were strong statistical predictors of the variance in binge-eating scores. Seasonality and addictive personality traits also accounted for a significant proportion of the variance in the measure of addictive behaviors. Conclusions are discussed in the context of brain reward mechanisms, motivational alternations in response to chronic over-consumption, and their relevance for the treatment of excessive appetitive behaviors.

## Introduction

The identification, classification, and clinical symptomatology of psychiatric disorders – even their very existence in some cases – are greatly influenced by prevailing social and economic factors. Consider, for example, the seemingly endless chronicle of *Hysteria*, which began many centuries ago, and then re-emerged in mid to late nineteenth century Europe as an exceedingly prominent syndromic study in medical science. It also garnered a great deal of public attention at the time (4). Hysterical symptoms like paralyzes and anesthesias were generally thought to stem from “unacceptable sexual impulses that were repressed to avoid unbearable anxiety and [were then] converted into physical symptoms” [Ref. (5), p. 534]. Hysteria was also viewed entirely as a “woman’s disease” whose cause was mostly impugned on the dominant Victorian morality of sexual prudery and constraint.^1^ A century later, Hysteria, as a discrete psychiatric entity, was largely discredited, and removed from the *Diagnostic and Statistical Manual* (DSM III) in 1980 (7). Soon thereafter, recognition of two other, more modern “women’s diseases” began to emerge. Like Hysteria, the establishment of Seasonal Affective Disorder (SAD) and Binge-Eating Disorder (BED) as legitimate psychopathologies has been shaped by contemporaneous sociocultural and environmental pressures, and anchored to historical events and contingencies.

Although seasonal mood disturbances had been recognized for millennia, SAD was not formally described and labeled until 1984 (8). A short time later, it was introduced in the DSM-III-R (9) as a “seasonal pattern” modifier to be applied to recurrent mood disorders like major depressive disorder. In addition to the rumination and negative affect seen in all mood disorders, SAD was pre-eminently characterized by pronounced vegetative symptoms – the so-called “hallmark” physiological signs such as anergia, increased appetite, and weight gain – that emerge in the autumn/winter and begin to remit in the spring (10). Many individuals with SAD also experience pronounced food cravings and frequent incidents of *binge eating* during the winter months (11, 12). Notably, however – and unlike other forms of unipolar depression – SAD occurs predominately in premenopausal adult women, and those working outside the home (13, 14). Most authorities agree that SAD reflects the high end of a normal human trait – *seasonality* – which describes the mood and behavior changes that occur across the seasons (15) and are manifest most prominently among those living in the temperate regions of the world. For thousands of generations, fluctuating patterns of food availability characterized the life of early *hominins* in these regions, and likely induced selective pressures on the genes responsible for food intake and energy conservation (16).

By the early 1990s, clinicians had also begun to recognize other patients with compulsive-overeating problems whose symptoms were quite similar to those seen in bulimia nervosa, but with the marked difference that their binge-eating episodes were not followed by any calorie-sparing or inappropriate compensatory behaviors (17). In other words, their binge eating occurred in the absence of hunger and deprivation, and typically involved the ingestion of large quantities of sweet and fatty foods in a relatively short period of time, with a strong feeling of loss-of-control over their food intake. This newly identified syndrome (BED) was known early on to have strong links with obesity (18) and mood disorders (19). Those with BED also display a more negative pattern of everyday emotions compared to their non-binging counterparts (20). BED was quick to make its way into the newly revised DSM-IV (21) in the *Eating Disorder Not Otherwise Specified* category, and soon afterward was listed in the Appendix of the DSM-IV-TR (22) as “a diagnosis for further study.” Twenty years later – in the recently released DSM-5 (23) – its status has been elevated to a fully recognized mental illness. Especially notable, and similar to the pronounced sex-bias in the prevalence of SAD, is evidence that lifetime prevalence of BED is estimated to be about twice to six-times more common in women (depending on the country surveyed), and to occur more frequently in mature adult women than in those of younger age (24).

**Figure 1:**
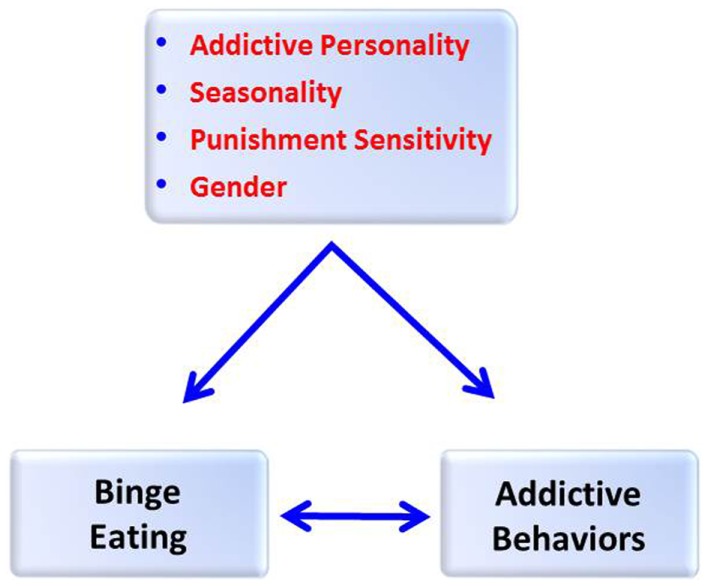
**A model predicting that addictive personality traits, seasonality, punishment sensitivity, and gender will be positively associated with a quantitative measure of binge eating and a frequency measure of addictive behaviors, and that binge eating and addictive behaviors will also be positively correlated**.

In this paper, it is proposed that high seasonality is a risk factor for chronic and compulsive overeating, especially in those characterized by anxious- and stress-prone personality traits – associations that could only occur in an environment with a superfluity of, and easy access to, highly palatable food resources. Given the well-established links between binge eating and addiction disorders [Ref. (1–3) for reviews], it is also suggested that seasonality, together with the same high-risk psychological profile, exacerbates the likelihood of engaging in a broad range of addictive behaviors (see Figure 1). While it is widely agreed that the development of clinically relevant psychopathologies depends considerably on factors unique to the individual, these are almost certainly aggravated by relevant environmental pressures and circumstances. The next section will describe some salient aspects of the sociopolitical landscape in Western nations during the time period when severe seasonal depression and chronic binge eating were declared “mental illnesses,” and which likely contributed to the emergence of, and impairment caused by, the troubling symptoms of these conditions. Following that, evidence-based inter-connections among seasonality, binge eating, and addictive behaviors will be reviewed. The penultimate section of the paper provides a description of, and empirical support for, the model proposed in Figure 1.

## Sociocultural and Environmental Influences

A few decades ago, a groundswell of striking political and economic events occurred that coalesced temporally, and played a significant role in the two emerging psychiatric disturbances described above – SAD and BED. They did so mainly by providing a social backdrop, which interfaced mal-adaptively with certain individual (risk) characteristics. For example, a global surge in the prevalence of obesity began – almost concurrently in most high-income countries – in the 1970s and 1980s (25), and continued to rise steadily during the following decade (26). Illustrated by recent data from a wide range of nations, representing considerable diversity in economic development, it is especially striking that there is now a 50% greater prevalence of obesity in women than in men (27). There is also some evidence that for women, but not men, the prevalence of obesity increased the most in the years from 1986 to 1994 (28). While many factors have contributed to the population weight gain, the most proximal include a reduction in the time-cost of production and transportation across the global food system (25), and the markedly “toxic” food environment exemplified by the caloric density and hyper-palatability of the processed products that comprise a substantial proportion of the modern diet (29).

Another impactful social phenomenon – occurring at about the same time – was the tremendous influx of women into the workforce (30). By the early 1980s more than half the (pre-retirement aged) women in the US were working outside the home. And, the large majority of these were married women in their middle adult years who were engaged in full-time employment (31). While this “remarkable revolution” (32) brought considerable economic empowerment to women – never experienced before to such a degree – it also came at a personal and social cost. The concurrent and accelerated increase in the elderly population resulted in working women often being the principal caregivers to dependent and aged parents as well as to their own children, and in some cases grandchildren – an occurrence of considerable burden and responsibility for these “women in the middle” (33). Moreover, divorce rates hit 50% for the first time in the early 1980s and women typically bore the brunt of single parenthood (34).

The 1980s saw the burgeoning of other social trends that had a considerable impact on women, and their psychological well-being. The iconic image of women’s bodies decreased significantly in size in the 1980s and 1990s, as depicted, for example, by the fashion media and the number of magazine covers showing full-body shots of women (35). A related development also occurred in the 1980s with the emergence of the commercial market of the women’s fitness and exercise industry – spearheaded by Jane Fonda and her workout books and videos. The “home exercise” media gave women a glamorized approach on how to burn calories and sculpt muscles, and had a great impact on forming public opinion about women, their bodies, and the virtues of ultra-slenderness (36). By the mid 1980s, the female readership of media pertaining to diet and exercise outnumbered that of men by a ratio of about 2:1 (36).

### Impact on modern “women’s diseases”

It is not difficult to see how the dramatic social, economic, and environmental changes that occurred in the last few decades of the twentieth century have played a role in the emergence of modern “women’s diseases” like BED and SAD. For instance, one of the most incompatible aspects of seasonality for the modern women is that increased appetite and food cravings are highly undesirable occurrences in societies that place a high value on an ultra-thin body ideal (10). These physiological changes are especially problematic and likely to contribute to substantial weight gain in the modern food environment with its superfluity of highly caloric and hyper-palatable foods. Another is that the time-and-energy demands on women who work outside the home, and who are also caregivers to children and elders, are elevated in a previously unprecedented way. Such roles are greatly incompatible with the anergy and social withdrawal that characterizes those with high levels of seasonality.

Consequent on the exigencies that compete for their time and energy, some vulnerable women experience high levels of stress in the face of inadequate resources to cope adaptively. Sex differences in the psychobiology of stress are now well-established with females producing a stronger HPA response than males. Individual differences in progesterone (P) levels in women appear to be one factor mediating this relationship. For instance, high P levels have been associated with lower stress and cue-induced craving, anxiety, and cardiovascular reactions (37). This effect may occur because P is a potent positive enhancer of GABA receptors, and GABA is an inhibitory neurotransmitter which diminishes dopamine, reducing stress responses, drug reward, and drug cravings (37).

We have learned that stress is strongly linked to binge eating, both as an initiating and as an exacerbating factor (38, 39). Evidence also suggests that different kinds of stressors produce distinct effects on food intake in females – specifically, that psychosocial adversity tends to augment consumption of highly palatable food (40), while interoceptive stress has the opposite effect (41). It is therefore relevant that women tend to display a greater preference for sweet foods compared to their male counterparts (42) – similar to the preclinical findings in male and female rats (43) – and they are more likely to experience food cravings (44). Research has also consistently shown that chronic intake of highly palatable food can produce dopamine signaling changes in the brain which promote binge-like patterns of consumption over time (45). In other words, the easy availability of hyper-palatable food, which has occurred during the period of rapid increases in obesity rates, is an important environmental precursor of BED. Not surprisingly, therefore, binge eating is significantly greater in women than in men (46), and the most pronounced increases in morbid obesity have also occurred in women (47). In a food environment with a superfluity and ubiquity of sweet and fatty cuisine, and in a social environment with immense emotional and time demands, it is not difficult to understand the emergence of compulsive-overeating behaviors prompted both by physiological factors, and by the use of “comfort food” to self-medicate a disturbed affect (48).

Stress is also strongly implicated in the development and perpetuation of drug and alcohol use and abuse, and has a critical role in the risk for relapse in addicted individuals (38, 49–52). While historically drug addiction was more prevalent in men than in women (53), there is evidence that the gap is narrowing, and that earlier disparities may simply reflect variation in opportunity and gender-role expectations rather than differences in vulnerability (54, 55). Indeed, many addiction risk factors appear to be more pronounced in women than in men. For instance, women tend to increase their rate of drug consumption more quickly than men, are more likely to relapse, and to have longer periods of drug use before their next attempt to quit (56, 57). Women with addictions also report more pronounced cravings and subjective drug effects than their male counterparts (58).

## Binge Eating, Addictive Behaviors, and Seasonality

In considering the relationship between binge eating and addictive behaviors, there are various possible causal routes of association. For instance, from a clinical and a psychobiological perspective, BED may reflect an eating-related manifestation of some underlying pathology that is shared among all addiction disorders. Inherent in that perspective is the precept that BED is an “addiction to hyper-palatable food.” Indeed, addiction-related processes are relevant to BED since this disorder has been associated with enhanced activation of brain reward pathways (59, 60) and with loss-of-control intake (61). On the other hand, BED and addiction disorders may simply be co-morbid conditions like schizophrenia and smoking in the manner that one syndrome serves as a risk factor for the other. Alternatively still, there may be other lurking influences, with significant causal links both to BED and to addiction disorders, which contribute to, or even account for, their co-occurrence. In this section of the paper, research related to these various viewpoints will be reviewed, including their conjoint association with seasonality.

### Common underlying risk factors

Some claim that current views on risks for mental illnesses have largely ignored the impact of the social and emotional world in which we currently live. Scheff (62), for example, has argued that modern Western society is built on a base of individualism, which has resulted in the “suppression of the social-emotional world in favor of thought and behavior” (p. 88). He explains that shame is one of the master emotions, but that the social alienation pervading our societies has created a forceful taboo on shame – in other words, we learn to be “ashamed” of our shame. The role of shame in BED is well-established. Indeed, it is integrated in the DSM diagnostic criteria for this disorder as “feelings of guilt, embarrassment, or disgust and [the individual] may binge eat alone to hide the behavior.” Other clinical research found that various negative emotions related to, and including, shame had the highest probability of leading to binge-eating behavior (20). A recent experimental study also demonstrated that individuals who reported feelings of shame found a buffet meal more desirable and were more likely to binge eat (63). They also ate more than control participants in a comparative taste test. Relatedly, shame-proneness has shown replicable relationships with drug use and abuse, largely as a means of coping with negative affect states (64, 65).

The risk for developing stress-related behavioral disorders like BED and addictions are also exacerbated in those who are less able to endure the subjective feelings of negative mood states (66–68). Low *distress tolerance* is a complex trait that reflects several characteristics including a lack of persistence with tasks that elicit psychological or physical discomfort, and the strong desire to do anything to prevent or stop feeling upset (69). It is seen as an emerging risk factor for various forms of psychopathology – most notably addiction disorders – and has considerable conceptual overlap with constructs like *harm avoidance* and *punishment sensitivity*. In turn, personality characteristics clearly interact with powerful environmental factors such as availability, cost, legal sanctions, and a host of attitudes and expectancies, which tend either to foster or to inhibit the use of addictive behaviors.

While emotional and personality traits can clearly be learned and shaped by environmental forces, they also have a pronounced biological basis to their development. Evidence from population-based transmission studies suggests that *common* genetic factors and physiological processes contribute to the abuse of a wide array of addictive substances (70). For instance, an inability to change one’s behavior when it leads to aversive consequences is a symptom common to all addiction disorders, and tends to reflect medial prefrontal cortical processes that are not functioning optimally (71). Correspondingly, such maladaptive responding is a key characteristic of those who suffer from compulsive overeating (72). Indeed, there is a rich body of evidence suggesting that BED is a specific phenotype of obesity with increased food-related impulsivity (59, 73) and executive-functioning deficits contributing to poor problem solving and decision making, and to cognitive inflexibility (3, 74).

Addictive behaviors also tend to be linked temporally and typically do not occur in isolation across the lifespan (75). It is relevant, therefore, that BED is associated with substantial psychiatric co-morbidities, especially those related to substance abuse and mood and anxiety disturbances (76). There is also good evidence that excessive food consumption and substance abuse both vie for similar neural pathways, and both cause similar reward dysfunction via receptor down-regulation in the dopamine pathways (77).

Importantly, and based on data from a family history study of the relatives of women with BED, it was concluded that virtually all the disorders co-morbid with BED followed a pattern of *independent* transmission except for substance abuse, in which the transmission pattern indicated a *shared etiology* with BED and a common mechanism of vulnerability (78). However, BED patients with a parental history of substance abuse appear to be a distinct sub-type of the disorder with a developmental profile characterized by an earlier age of onset and a more rapid progression. They are also more likely to have started binge eating before they began to diet, and more likely to meet the diagnostic criteria for a mood disorder (79). Other etiologic links have been demonstrated between overeating and alcohol use. For instance, women with a family history of alcoholism had almost double the odds of being obese compared to those without a family history – a statistical association that remained robust after controlling for a number of well-established covariates of obesity (80). While this relationship was seen in men as well, it was not as strong as in women.

### Addiction to hyper-palatable foods

An increasing number of researchers and clinicians now subscribe to the view that compulsive-overeating shares explicit similarities with substance-abuse disorders, and therefore can be conceptualized as an addiction – at least in some cases (2, 3, 81, 82). Initially, the *food-addiction* concept earned its scientific credibility from compelling preclinical evidence that a syndrome remarkably similar to drug dependence can be very successfully induced in animals when they are given access to sugar (83). Rodent studies of food addiction have burgeoned in the past decade, and typically use behavioral paradigms based on experimentally inspired analogs of the DSM-IV-TR (22) criteria for substance dependence to demonstrate pronounced behavioral and physiological similarities between excessive drug use and diets high in sugar, fat, or both (1).

Clinical food-addiction research is sparser and more recent, and has relied, almost entirely, on studies using the *Yale Food-Addiction Scale* [YFAS] to establish classification (84). This assessment tool employs the DSM-IV-TR criteria for substance dependence as a template for diagnosis. To date, studies have found substantial diagnostic overlap or co-morbidity (approximately 50%) between BED and YFAS food addiction, in addition to many shared psychological and biological risk factors (1, 85, 86). An even greater overlap was found in an earlier study of women diagnosed with BED (87) where 92% of the sample met the DSM-IV-TR criteria for substance dependence when the word “food” was substituted for “drug” in the interview questions. A recent qualitative study of obese women with BED has also confirmed that a high proportion endorsed DSM symptoms of substance dependence when food was the “substance” in question (88). These women felt that “loss-of-control” overeating, the inability to stop this behavior despite strong wishes to do so, and extreme cravings were the characteristics of their disorder which most resembled an addiction. Importantly, they emphasized that it was only highly palatable “junk” foods that elicited these feelings. This last point endorses the criticism that “food addiction” is a poorly descriptive label for cases of compulsive and impairing overeating. More apt terminology may be *hyper-palatable food addiction* or *sugar/fat/salt addiction*.

### BED and food addiction: A continuum of severity

When assessing the established animal models of food addiction, an important observation is that a pattern of compulsive intake – behaviorally similar to what we call *binge eating* in the human condition – is an universal feature (1). In other words, animals do not display the cardinal symptoms of addiction disorders, without also showing binge-like consumption of the experimental food. Moreover, all animal models require the introduction of highly palatable foods – that is, compulsive intake does not occur in response to the bland chow animals are normally fed. Based on these preclinical findings, and also on the pronounced clinical and phenotypic overlap between those with a diagnosis of BED and those with a YFAS-diagnosis of food addiction, we have proposed that food addiction is not a separable entity from BED but rather that it reflects – at least in many cases – a more severe and more compulsive sub-type of BED. In a recent study, preliminary support was found for this hypothesis (89). We compared two equivalent groups of overweight men and women with BED – one *with* co-occurring YFAS-diagnosed food addiction and the other *without*. While the two BED groups were equivalent in age and BMI, those with food addiction were significantly more likely to overeat for emotional and cue-driven reasons, they had more severe binge eating and food cravings, and they were more responsive to the rewarding properties of food. This group also had more addictive personality traits, was more impulsive, and had greatly elevated symptoms of depression compared to their BED counterparts without co-occurring food addiction. These findings converge with a related study of food addiction in BED patients where the number of YFAS *symptoms* correlated positively with Beck depression ratings, poor self-esteem, and difficulties in emotion regulation, as well as with frequency of binge episodes (86). Similarly, in a study of weight-loss treatment-seeking adults, YFAS symptom scores related significantly to increased depression, increased emotional and binge eating, and to less weight loss after several weeks of treatment (90).

In our study (89), we also compared the BED group *without* food addiction to a weight- and age-matched group of adults *without* BED and *without* food addiction on the same variables described above. By contrast to the earlier comparisons, these two groups were remarkably similar to each other except, not surprisingly, that the BED group reported more frequent binge eating, elevated food cravings, and greater hedonic responses to food than the non-BED controls.

### Seasonality and addictive behaviors

Human biological functioning is profoundly dependent on the daily rotation of the earth on its axis, and its annual trek around the sun. Due to factors such as shift work, long work hours, extended commutes, and around-the-clock global communication, the recent imposition of a “24/7/365 culture” has been causally implicated in a broad range of pathologies including depression, insomnia, obesity, and immune impairment (91).

Neurons in the suprachiasmatic nucleus of the hypothalamus comprise the body’s so-called “master clock,” which regulates the temporal organization of many human behaviors like sleeping and wakefulness (92). As part of this process, the brain’s pineal gland discharges the hormone melatonin at night to promote sleep and other cyclical physiological events. The circadian system also fosters seasonal changes because of its ability to measure changing photoperiod across days of the year (93). In other words, we have an endogenous temporal program that uses daylight as an entraining cue (91). Across the population, however, there is great variation in photo-responsiveness with some being highly photoperiodic, others not at all, and a range of intermediate types (94).

Various family and genetic studies have demonstrated a biological link between seasonality/SAD and drug-abuse disorders like alcoholism (95, 96). For example, genes that have a regulatory role in the circadian pacemaker system have also been associated with the consumption, sensitivity, and abuse of alcohol (97). Other evidence also supports seasonal trends in the use of various addictive substances with significant peaks in the winter and a diminution in the summer months (98, 99). Relatedly, links between drug use and depression become stronger with increasing latitude (93). And finally, there is evidence that addiction may be more prevalent in those with a compromised “master clock” and/or with mood disorders that have a circadian basis (100). As was discussed earlier in this paper, there are also established links between BED and SAD. Interestingly, therefore, a study of Google internet queries across all major mental health disorders over a 5-year period from 2006 to 2010 followed a pattern of winter peaks and summer troughs, with these seasonal differences being most pronounced for disordered eating (101).

## A Process Model Linking Seasonality, Psychological Vulnerability, and Gender to Binge Eating and Addictive Behaviors

In summary, this paper has reviewed evidence linking binge eating and addictive behaviors, and has identified stable psychological traits including seasonality and proneness-to-stress as common vulnerabilities. To date, no research has assessed the cumulative risk potential of these factors for binge eating and other addictive behaviors. Figure 1 describes two models proposing, on the one hand, that addictive personality traits, punishment sensitivity, and high seasonality each contribute unique variance to symptom scores for binge eating, and on the other hand, that these variables would also contribute to the variance in a composite measure of addictive behaviors. Gender was added to both models to assess male-female differences. We also predicted that binge eating and addictive behaviors would be positively and significantly inter-correlated.

### Participants

A community-based sample of adult men (*n* = 103) and women (*n* = 321) between the ages of 25 and 50 years, and representing a broad range of body weights (BMI = 32.3; SD = 9.4), were recruited for the study. Participants were fluent in English and had lived in North America for at least 5 years prior to their enrollment. Exclusion criteria included a current diagnosis of any psychotic disorder, addiction disorder, a serious medical/physical illness such as cancer or heart disease, or the use of medication with a high probability of affecting appetite like stimulants or neuroleptic drugs. Participants were recruited from posters located at a variety of public institutions. Advertisements were also placed in local newspapers and various online sites. All measures were assessed individually in a hospital research laboratory. The procedures employed in this study were approved by the university Research Ethics Board, and were carried out in accordance with the Declaration of Helsinki. As an initial step in the screening procedure, a short telephone interview was carried out to confirm basic eligibility criteria before the in-person assessment appointment took place. During this appointment, height and weight were measured with participants wearing light indoor clothing and standing in stocking feet.

### Measurements

*Binge Eating* was assessed by 5-items of the *Binge-Eating Questionnaire* (102), which obtains information about frequency and severity of symptoms such as loss-of-control over eating, and negative affect following a binge. The alpha coefficient for this study was 0.85.

*Addictive Behaviors* were assessed by the *Shorter PROMIS Questionnaire* (103), a self-report instrument for the concurrent measurement of 16 addictive and/or excessive behaviors. The scale items reflect common characteristics of addictive behaviors such as using more than was intended and increased capacity or tolerance. For the purpose of this study, a total score was created by summing the items for the following six subscales: caffeine, recreational drugs, sex, nicotine, shopping/spending, and alcohol. Other subscales were deemed insufficiently related to conventional addiction disorders to be included. Only a subgroup of the sample (*n* = 211) completed this measure. The alpha coefficients for the six subscales ranged from 0.80 to 0.97.

*Addictive Personality Traits* were assessed by the 32-item *Addiction Scale* of the *Eysenck Personality Questionnaire-Revised* (EPQ-R) (104). This well-validated addiction-proneness scale was derived empirically by identifying items of the EPQ-R, which significantly differentiated those with drug addictions from normal controls. The selected items largely reflect impulsiveness, anxiousness, and negative affect. The Cronbach alpha coefficient in the present study was 0.79.

*Seasonality* was assessed by the *Seasonal Pattern Assessment Questionnaire* (SPAQ) (105). The Global Seasonality Score (GSS) is the sum of six items (0–4) asking about seasonal changes in sleep length, social activity, mood, weight, appetite, and energy. Higher scores reflect greater seasonality. The GSS is the most frequently used dimensional measure of seasonality and has demonstrated good psychometric properties (15). The GSS alpha coefficient for our study was 0.87.

*Sensitivity to Punishment (SP)* was assessed by 24 binary items of the *SP Sensitivity to Reward Questionnaire* reflecting the respondent = s avoidance responses under conditions of punishment (106). This sub-scale was developed to evaluate variation in a neurobiological motivational system – known as behavioral inhibition – alleged to underlie anxiousness and distress sensitivity. The alpha coefficient for the present study was 0.87.

The data were analyzed using stepwise regression procedures with punishment sensitivity, addictive personality traits, seasonality, and gender entered as independent variables. In the first model, binge eating was the dependent variable. Results demonstrated that the three psychological independent variables each contributed a significant proportion of the variance to the dependent variable, and that females had higher scores than males. Together these variables accounted for 26% of the variance in binge-eating scores. Summary statistics are shown in Table 1. In the second analysis, the addictive-behaviors measure was regressed on the same four independent variables. Only addictive personality traits and seasonality were significant predictors in the model, and together accounted for 19% of the variance in the dependent variable (see Table 2). Finally, and as hypothesized, the correlation between binge eating and addictive behaviors was highly statistically significant and of virtually identical magnitude in women (*r* = 0.43) and men (*r* = 0.45). Collectively, these findings support the conjoint relationship between binge eating and the use of other addictive behaviors, and they endorse the view that seasonality and addictive personality traits – the latter of which reflect elevated levels of emotional reactivity, proneness-to-stress, impulsivity, and negative affect – are common risk factors for both potentially pathological behaviors.

**Table 1:** **Hierarchical regression analysis summary (*n* = 412) for the independent variables (punishment sensitivity, addictive personality, seasonality, and gender) predicting the dependent variable (binge-eating symptom scores)**.

Variable	*B*	SEB	*t*	*p*	*R*^2^ change
**Step 1**
Punishment sensitivity	0.14	0.02	9.73	<0.0001	0.187
**Step 2**
Punishment sensitivity	0.09	0.02	5.15	<0.0001	
Addictive personality	0.09	0.02	4.85	<0.0001	0.044
**Step 3**
Punishment sensitivity	0.09	0.02	5.03	<0.0001	
Addictive personality	0.08	0.02	3.88	<0.0001	
Seasonality	0.05	0.02	3.06	0.002	0.017
**Step 4**
Punishment sensitivity	0.08	0.02	4.72	<0.0001	
Addictive personality	0.08	0.02	4.13	<0.0001	
Seasonality	0.05	0.02	2.99	0.003	
Gender	−0.52	−0.19	−2.76	0.006	0.014

*Total *R*^2^ = 0.26*.

**Table 2:** **Hierarchical regression analysis summary (*n* = 211) for the independent variables (punishment sensitivity, addictive personality, seasonality, and gender) predicting the dependent variable (addictive behaviors)**.

Variable	*B*	SEB	*t*	*p*	*R*^2^ change
**Step 1**
Addictive personality	2.93	0.45	6.50	<0.0001	0.167
**Step 2**
Addictive personality	2.58	0.47	5.48	<0.0001	
Seasonality	1.07	0.46	2.32	0.021	0.021
**Excluded variables**
Gender			1.00	0.319	
Punishment sensitivity			0.20	0.839	

*Total *R*^2^ = 0.19*.

## Conclusions

In the opinion of this author, the evidence is irrefutable that compulsive overeating – what we call BED in some cases, or food addiction in others – is very similar to what is understood by terms like “drug abuse” and “substance dependence.” These are all labels which acknowledge that chronic over-consumption of rewarding substances, and thereby excessive stimulation of a core mechanism of human survival – the brain’s common reward pathway – diminishes our ability to function with full capacity for reason and free will. What we choose to call this dysfunction is perhaps less important than finding appropriate treatment and restorative measures for individuals who suffer from the condition.

All psychiatric disorders, including drug abuse and behavioral addictions, have a relatively low base-rate occurrence, even though the related symptoms exist along a continuum of severity across individuals in the population. Therefore, most people who use addictive substances – drugs or highly palatable food – do not develop dependence. Nevertheless, gaining a better understanding of the sequelae of behaviors such as overeating is highly pertinent because these activities are known to cause alterations in brain reward functioning and to reduce sensitivity in these pathways. They are also highly likely to cause weight gain over time. Developing health-related behavioral strategies that recognize changes in motivation as a consequence of excessive food consumption are also important because of evidence that brain neuro-adaptations can increase the likelihood of using and abusing other addictive substances (107). Avena and her colleagues (107) have argued, for a number of reasons, that such treatment approaches are particularly important for the growing cohort of middle-age and elderly adults in the population. First of all, a reduction in dopamine reward functioning is a normal process of aging (108). Additionally, rates of obesity and of substance abuse are currently on the rise in this group, suggesting that these individuals may be at greater risk for developing a dependence on rewarding substances than are younger adults. From a treatment perspective, it is also important to take account of seasonal variation in the symptoms of binge eating and addictive behaviors in order to provide appropriate coping strategies for those who have pronounced negative changes in mood and behavior during the dark months of the year.

## Conflict of Interest Statement

The author declares that the research was conducted in the absence of any commercial or financial relationships that could be construed as a potential conflict of interest.

## Footnotes

^1^Interestingly, some have argued that the prim sexual mores of the Victorians were merely “uttered” sentiments and imitated largely for the sake of appearance, while their actual sexual behaviors – at least among the privileged classes – were marked by considerable freedom, and even license (6).
